# Associations between Social Isolation, Loneliness, and Cognitive Frailty in Older Indians: Variations by Gender and Place of Residence

**DOI:** 10.1002/alz70858_107328

**Published:** 2025-12-26

**Authors:** Thalil Muhammad, Manacy Pai, Madhurima Sharma

**Affiliations:** ^1^ Pennsylvania State University, State College, PA, USA; ^2^ Kent State University, Kent, OH, USA; ^3^ International Institute for Population Sciences, Mumbai, Maharashtra, India

## Abstract

**Background:**

The potential associations between “social health” and cognitive frailty is less explored. An inquiry of this nature would be particularly relevant in the Indian context, where family structures and, consequently, family dynamics are continuing to evolve, and income inequalities can greatly limit or improve health outcomes. In this study, we examined the associations between social isolation, loneliness, and cognitive frailty among community‐dwelling older persons in India. Moreover, we explored whether these associations vary by gender and rural‐urban residence.

**Method:**

Data are from the Longitudinal Study of Ageing in India (LASI), which included 31,464 older adults aged 60+. Social isolation was measured using indicators like marital status, social group membership, and social engagement. Loneliness was assessed using a single question on feeling alone. Cognitive frailty was defined as the co‐occurrence of cognitive impairment and physical frailty. Multivariate logistic regression models were used to analyze the data, adjusting for socio‐demographic, health, and lifestyle factors.

**Result:**

Social isolation and loneliness were common, affecting 38.8 and 37.2% of participants, respectively, with higher rates among women and rural residents. Cognitive frailty was more prevalent in socially isolated and lonely individuals. The association between social isolation and cognitive frailty was stronger than the linkage between loneliness and cognitive frailty. Gender differences showed that the odds of physical frailty (AOR: 1.23; 95% CI: 1.01‐1.35), cognitive impairment (AOR: 1.72; 95% CI: 1.57‐1.90), and cognitive frailty (AOR: 1.50; 95% CI: 1.28‐1.75) were elevated in socially isolated women. Rural residents with social isolation (AOR: 1.63; 95% CI: 1.40‐1.90) and urban residents with loneliness (AOR: 1.51; 95% CI: 1.11‐2.56) had significantly greater odds of cognitive frailty compared to their peers.

**Conclusion:**

Social isolation and loneliness are key factors linked to cognitive frailty in older Indians, with significant variations by gender and place of residence. Addressing these social challenges, especially in rural areas and among vulnerable groups, is crucial for promoting healthier aging. Future research should explore how these associations change over time and identify effective interventions.

